# Selections and Abstracts

**Published:** 1916-04

**Authors:** 


### SELECTIONS AND ABSTRACTS

### THE APPLICATION OF ANOCI ASSOCIATION TO OBSTETRICS

(By Carl L. Hoag, M. D., San Francisco.)

The present furor about "twilight sleep" has brought great pressure to bear upon the conscientious physician who does not feel justified in using a procedure which entails such risks. Recognizing the general demands for the more extended use of anesthetics in labor, he will doubtless welcome any method which secures this end without too much danger to mother or child. This consideration has prompted me to report the following procedure without awaiting the accumulation of a large number of cases.

As will be seen, the method is not new in itself but is the application of a now well accepted principle to this new field of obstetrics.

Three years of trial in the surgical field has firmly established the efficiency of anoci association in the reduction of shock and distress, with a corresponding decrease in the time of convalescence. It has already done much in removing the fear of operative procedure from the lay mind. The principle objection has come from the surgeon because muscular relaxation under nitrous-oxide-oxygen anesthesia is very much less complete than that previously secured by the use of chloroform or ether. Only those have achieved the best results who have fully realized that the nerve blocking accompanying this light anesthetic must be fully as complete as that necessary for operations under local anesthesia alone.

During the past year nitrous-oxide-oxygen analgesia has been used in obstetrics by a number of men—notably Webster and Lynch of Chicago—with marked success. No untoward effects have been noted on either mother or child. Lynch states that uterine contractions are usually increased. He makes no statement, however, as to the degree of relaxation of the perineum as compared to that usually secured when chloroform is used If there be any serious objections to the use of nitrous-oxide-oxygen anesthesia in labor it will probably arise at this point. After our experience in abdominal surgery we cannot expect to see that relaxation of the vaginal outlet which is so essential in preventing severe lacerations. It occurred to me that the same cocainization of the muscles which brings about adequate relaxation of the abdominal wall when anoci association is well carried out should not only overcome this objection but might bring about a distinct advance in obstetric practice. Just as the local anesthesia has made the use of gas-oxygen possible in surgery, the same procedure may make it the anesthetic of choice in labor.

The following case will illustrate the feasibility and distinct advantage of this method:

Mrs. T., aged 22, primipara, confined April 11, 1915, when three weeks overdue. She was a well developed, well nourished woman and labor progressed normally; first stage nine hours, second stage forty-five minutes. Toward the end of the first stage nitrous-oxid and oxygen analgesia was begun by Dr. Harry Tuckey, using a new Teter apparatus. The gas was shut off between the bag and nose piece between pains, so little was lost. Experiencing no pain, the patient worked very well and absolutely at my direction. Just before the head began to dilate the perineum, I turned back the vulval edges and injected the levator ani muscles and perineal body very thoroughly with one-half of one per cent. novocaine. About 20-30 c. c. were used. The effect was striking. The outlet soon relaxed and became very flabby, and when distended by the oncoming head (R. O. A.) dilated readily without the least sense of pain or the increased effort on the part of the patient that we usually see. The head was delivered as easily as if she had been under the surgical degree of chloroform, and without other than a small mucous laceration. The oxygen was then immediately turned on and, as the cord was still pulsating, the baby—then slightly cyanotic—cleared up like magic, became very red and began to cry spontaneously. The placenta was delivered by the Crede method, likewise under gas.

About 100 gallons of nitrous-oxid and 30 gallons of oxygen were used over a period of one hour. The amount of novocaine was about one grain. Many times that amount is not toxic and could have been used if necessary to secure complete relaxation.

The striking points are:

(1) The delivery without pain, thus insuring better cooperation between the patient and accoucheur.

(2) The greater efficiency of the uterine contractions which shorten the time of labor.

(3) The absence of muscular spasm and rigidity of the perineum due to the blocking of pain reflexes by the local injection, thus permitting slow and gradual dilation under perfect control. This great advantage cannot be secured by general analgesia alone.

(4) The fact that oxygenation of the mother has an immediate effect upon the child, clearing up any cyanosis that may be present.

Local infiltration of the perineum, besides securing relaxation and anesthesia, also allows of episiotomy at any moment when a laceration seems unavoidable. Following delivery, this incision or any laceration that may result, can be painlessly repaired without prolongation of the general anesthetic.

The anesthetic effect of novocaine is transcient and is usually maintained only for thirty or forty-five minutes. In prolonged cases either a secondary blocking can be done when the head recedes, or the original injection of novocaine can be immediately followed by quinine urea as in surgical anoci.

The effect of nitrous-oxid and oxygen on the child has been too well tested by Webster, Lynch and Woodyatt to need discussion here, but we know much less about the effect of novocaine upon the fetus. The amount actually absorbed and reaching the child through the blood stream prior to delivery must be very small and a quantity not greater than that safely used in numerous cases of Cesarian section under local anesthesia.

Whether injection of the cervix will prove of sufficient advantage to justify the added risk of infection is doubtful. At present it does not seem advisable.

There are no dangers in infiltration of the perineum from sepsis or otherwise. The needle is inserted through the vaginal mucous membrane which offers a practically sterile field. Hemstome seldom, if ever, develop. Any edema produced by the solutions is quickly absorbed. Repair of tissues is not retarded.

In conclusion, it is my belief that the use of anoci association, that is, the combined use of nitrous-oxid-oxygen analgesia with local infiltration of the perineum, offers a safe and efficient method of conducting labor. It is a logical procedure founded on established principles, apparently free from danger to either mother or child and sufficiently simple to be within the scope of general use.

### EXTRACTS FROM AN ADDRESS DELIVERED IN CHICAGO, DECEMBER 10, 1915, BEFORE THE EUGENICS EDUCATIONAL SOCIETY BY CASPER L. REDFIELD.

It has been said frequently that each man is the product of his environment. In a measure that is true, but no environment will make a Shakespeare out of an ordinary man. A great man is born, which means that he is the product of a particular kind of breeding. Similarly, the feeble-minded man is born, which also means that he is the product of a particular kind of breeding The kinds of breeding which produce our great men and our feeble-minded men are as widely separated as are the men themselves. I cannot, in the time at my disposal, go into all of the intricacies by which different kinds of men are produced by breeding, but I can give some of the main essentials, and from these you can obtain a fairly clear understanding of what it is that leads toward improvement, and what it is that leads toward degeneracy.

They tell us that man and the other higher animals have evolved from lower forms of animals by selection, but those who make that statement overlook a very obvious absurdity in their claim. To have selection, parents must have offspring, and to have more selection the offspring must produce another generation, and these in turn another. Each generation gives opportunity for selection, and the more generations the more selection. Anything which would reduce the number of generations in a given period of time would reduce the opportunities for selection to accomplish anything.

At some time in the past there was a common ancestor for man and the higher apes. There have been less generations, and consequently less selection, in the line leading from that common ancestor to man, than in the lines leading to the apes. Further back in the past there was a common ancestor for the higher apes and the lower monkeys. There have been less generations, and consequently less selection in the lines leading to the higher apes than in the lines leading to the lower monkeys. Extend that examination to the different species of active animals and you will find that each advances from a lower to a higher stage involved the elimination of selection, and that the actual advance has been inversely proportional to the amount of selection. Carried to its logical conclusion this means that the greatest possible advance will occur when selection is reduced to zero.

They tell us that acquired characters are not inherited, but I am telling you that the persons that make that statement never investigated the matter and know nothing whatever about it. They simply repeat what they have been taught, and they cling to the dogma because it agrees with their preconceived ideas. The theory that acquired characters are not inherited originated in a misconception of what an acquired character is, and in an experiment which is absurd on its face.

To acquire means to obtain by effort, by exertion, by the performance of work. An acquired character is one obtained by exercising an organ, or by the work performed by the organ. It consists of a physiological change occurring within the organ which is dynamic in character and is called dynamic development. The amount of an acquirement is proportional to the amount of work performed. A mentally active man has a better developed brain at the age of fifty than he had at the age of twenty, and the difference is due to the extra amount of mental work performed.

If an acquirement is to be inherited, the parent must make the acquirement first and get the offspring afterwards, not get the offspring first and make the acquirement afterwards. Of those who deny the inheritance of acquired characteristics, what one ever took this into consideration and compared the progeny of parents of different ages on the basis of acquirements? Not one. They have failed to take even the first step in such an investigation whereas they should carry such a one through three or four generations of ancestors.

Among certain eugenists there is a theory that it is impossible to produce an individual which is superior to anything which previously existed. That is, if some very superior individual exists it is because there was, somewhere in his ancestry, a similar superior individual. This theory amounts to a denial of evolution and a return to the Garden of Eden story with Adam and Eve originally created equal to any individual who has since existed.

It is not clear how widely extended this theory is, but it seems to be back of the proposition to sterilize a large part of the population. That proposition is a public confession, by those who make it, that they know absolutely nothing about what causes improvement and what causes degeneracy. In their despair at seeing no way to improve the race other than that of killing off the inferior, they propose the killing process by indirection.

It seems never to have occurred to these gentlemen to write out the pedigree of some remarkable individual for three or four generations and then examine that pedigree for the purpose of learning if there was anything remarkable about the way he was produced. Wedded to a preconceived theory which they are anxious to support, they make statements without stopping to consider what those statements mean when carried to their logical conclusion.

Let us consider the horse. A century ago there was no horse in the world capable of trotting a mile in three minutes. Now we have horses in the world capable of trotting a mile in two minutes. This is an absolute and very great advance in power made in the past 100 years. It has been said repeatedly that this improvement came about through selection, but the statement is not true and is made in complete ignorance of the facts. Selection has been used abundantly among horses, but that selection is not connected with the improvement which has taken place.

High speed at the trot is not a natural gait for horses. It is an artificial gait which never existed in any breed of horses until forced there by the art of man. Less than a century ago the only high speed gait for horses was the run, and when trotters were forced for speed they would break into a run. Now we have "born trotters" which will stick to the trot no matter how hard they are forced, and trotting speed approaches running speed. Here is a new character in the trotter of to-day.

To have selection a mare must have several foals. If she produces but one foal in her entire life, there can be no selection in her line. It is take that foal or none. Write out the pedigree of any 2:10 trotter, it matters not what one, and extend that pedigree to the time when there was no such thing in the world as a 2:30 trotter. In that pedigree there will be from five to twenty years, no one of which ever had more than one foal in her life. The other mares, and the sires in the pedigrees, will be found, on investigation to have produced less than the normal number of foals. Also, the lines of improvement to our high-speed trotters averages only seven generations to the century, while the normal number is ten generations. Actual improvement came in those lines in which opportunity for selection was reduced to its lowest limit.

The same thing is true in intellectual power in man. Take any list of intellectually eminent men and you will find that they were sons of men much older than the average age of fathers when sons are born. A few will be found to be sons of comparatively young fathers, but push the investigation in those cases a little further and you will find that while it is possible to get an eminent man from a young father, it is impossible to get one from a succession of young parents. A succession of young parents always results in the production of mental inferiority, and, if the parents are unusually young, in such a succession the product is weak mentality.

To maintain any group of animals on a level in its power capabilities there must be a certain amount of acquirement per generation before reproduction. If the amount of acquirement is decreased there is a decline in power capabilities toward a lower corresponding level. If the acquirement is increased there is a rise toward a higher corresponding level. The age of parents at time of reproducing is one factor in measuring the amount of acquirement, and an investigation which did not consider the factor in at least three generations of ancestors would be superficial.

### IN THE HOUSE OF REPRESENTATIVES

February 11, 1916.

Mr. Linthicum submitted the following resolution; which was veferred to the Committee on Rules and ordered to be printed.

RESOLUTION

Whereas it is reported by the Bureau of Animal Industry that ninety-four and five-tenths per centum of the creameries of the country are insanitary to a greater or less degree; that sixty-one and five-tenths per centum of the cream used is unclean or decomposed, or both; that seventy-two and six-tenths per centum of the cream is not pasteurized, but is made into butter to be consumed in a raw state, in which state disease germs retain their virulence for a long period of time; that a large percentage of all dairy cattle are affected with tuberculosis; and that infected dairy products are among the active agents in the spread of tuberculosis, typhoid fever, and other infectious diseases; and

Whereas dairy products are the most widely used of all human foods; and

Whereas dairies and dairy products are not subject to Federal inspection, so that there is a growing sense of alarm among the consumers: Therefore, be it

RESOLVED, That the Speaker of the House of Representatives appoint a committee of five members of the House whose duty it shall be to investigate and report as speedily as practicable (a) whether conditions prevailing in dairies and dairy products seriously menace the health and property of the people of the United States; (b) whether Federal inspection and supervision, either alone or in co-operation with State and municipal inspection and supervision is necessary to the reasonable protection of the health and property of the citizens of the United States; (c) if so, then the best and most economic methods of inaugurating and enforcing such inspection and supervision.

Second. That for the purpose of fulfilling its functions said committee is empowered to summon and examine witnesses, enforce the production of records, and to do all other things needful and lawful to accomplish its purpose.

Resolved further, That the expenses of said inquiry and investigation shall be paid out of the contingent fund of the House upon vouchers approved by the chairman of said committee, to be immediately available.

---

### MODERN METHODS OF URETHRAL INSPECTION.*

BY J. T WINDELL, M D. F. A. C S

LOUISVILLE, KENTUCKY.

Before considering in detail the modern methods of urethral inspection, a brief anatomical survey seems advisable. The male urethra is a musculo-membranous canal beginning proximally at the vesica urinaria, and terminating distally at the meatus urinarius externus. The average length is eight and one-half inches when the penis is flaccid, and the caliber varies considerably in different individuals.

While anatomically the urethra is divided into three sections, (a) the penile, (b) the membranous, and (c) the prostatic, but two divisions are recognized clinically, viz., (a) the anterior, and (b) the posterior. The former extends from the external meatus to the anterior layer of the triangular ligament, the latter (including both membranous and prostatic) from the triangular ligament to the urethro-vesical orifice. As the urethra is a collapsible tube its walls are in immediate contact excepting when artificially separated, e. g., during micturition, the emission of semen, intra-urethral instrumentation, the presence of foreign bodies, etc. The penile portion is mobile, distensible, readily yielding to pressure and change of position; whereas the permanently curved remainder is less distensible and normally fixed in its position by rigidity of the perineal structures. The average length of the anterior section is six and a half inches, the posterior about two inches. There are two constrictions, three dilatations, a movable, and a fixed curve. The constrictions are (a) near the external meatus, and (b) near the internal vesical sphincter; the dilatations (a) at the fossa navicularis, (b) at the bulb, and (c) in the mid-portion of the prostatic section; the fixed curve extends backward from about half an inch distal to the bulb; the movable curve is embraced in the penile portion.

The urethral mucosa is continuous throughout the length of the canal, its epithelial lining consists of columnar cells excepting at the distal orifice where the cells are squamous, and is arranged in longitudinal folds in so far as the membranous and penile sections are concerned. There are numerous glands situated within the submucosa and the connective tissue, the orifices of which are found in the mucosa. These openings vary in size and are directed distally, one much larger than the remainder (lacuna magna) being situated upon the dorsal surface of the fossa navicularis about half an inch internal to the meatus. The prostatic portion is the widest, and has a narrow longitudinal ridge (verumontanum) three-fourths of an inch in length upon the dorsal wall. There are fossæ upon either side of the verumontanum, the uterus masculinus occupies the distal portion, and the orifices of the prostatic glands are found in the fossæ.

It is probable that urethral pathological lesions, together with their various complications and sequelæ, antedate the written history of mankind, since the prevalence of "the running issue out of the flesh" is mentioned in the oldest historical records. It was quite natural, therefore, that ancient practitioners of medicine should devise crude instruments and methods of procedure by which it was hoped the urethral interior might be successfully explored. Catheters and urethral sounds are among the oldest known surgical appliances, and en passant the observation is pertinent that some of the indications for their employment are as imperative today as they were centuries ago.

The modern urologist, equipped with the latest instruments of precision by means of which diagnostic and therapeutic accuracy may be assured, can scarcely realize the difficulties under which the older practitioners labored in the diagnosis and treatment of urethral lesions. While an existing urethrostenosis might be temporarily relieved by the introduction of crudely constructed sounds, and an overdistended bladder successfully emptied by the insertion of a catheter or other hollow instrument, or by suprapubic puncture, the etiology, pathology and bacteriology of urethral lesions being then imperfectly understood, necessarily the diagnosis remained uncertain and treatment was doubtless oftentimes grossly misapplied. When a more comprehensive understanding of urethral pathology made possible greater accuracy in diagnosis, some of the former therapeutic difficulties were overcome; but there still remained no method of procedure by which the urethral mucosa could be exposed to visual inspection until invention of the urethroscope about the middle of the last century.

The first attempt to visually examine the urethral interior through an endoscopic tube was made by Desormeaux. He used a crude instrument in which the source of light was a common lamp, the rays being reflected into the urethra with ordinary mirrors; later Grunefeld reflected the light rays by means of a head mirror. Various modifications and improvements in style and construction of the instrument were afterward made by Casper, Otis, Fenwick, Posner and many others. Oberlander and Nitze (1877) first successfully used the electric current (with a platinum loop) for illuminating the urethral interior. For the first really practical endoscopic lamp, however, we are indebted to Valentine.

Until 1894 the straight tube was employed for examination of both the posterior and anterior urethra; its introduction through the posterior canal was sometimes accomplished with considerable difficulty, and in some instances serious injury was inflicted upon the delicate urethral mucosa. Lowenhardt then devised an instrument having a "posterior urethral curve" to be used without dilatation with either air or water. To Goldschmidt (1906) and Wossidlo (1907) are due the credit for having made the greatest discoveries in posterior urethroscopic investigation. Their instruments permit examination of the posterior urethra under either water or air dilatation. The most efficient urethroscopes now in popular use are those of Goldschmidt, Wossidlo and Buerger. With either of these instruments the entire urethral interior may be satisfactorily inspected, and where desired therapeutic applications and surgical procedures may also be successfully executed under actual visaul control.

Quite recently Gordon has devised and described an examining and operating instrument which he states is a combination of Wyndham Powell's English, Koch's American, and Luys' French urethroscopes, for use in the anterior urethra with or without air dilatation. The lamp is so protected that it cannot become soiled nor interfere with cleansing the urethra, and the field is magnified. Where the meatus will admit the open tip of the instrument an obturator is unnecessary; after being inserted about one-fourth of an inch, the lens is adjusted, the lamp lit, and the patient requested to "press the dilating bulb." Unless considerable stenosis is present it is claimed the urethra dilates and its walls may be examined in advance of the tube which is gradually introduced to the external sphincter. If at any time during the examination a non-dilated "rosette picture" is desired, it is only necessary to withdraw the plug connecting the air bag, and the urethra collapses.

Under certain circumstances visual inspection of the urethral interior is just as essential as minute examination of other portions of the human organism which may be involved in definite pathology. Practically all the former difficulties in connection with urethroscopy have been overcome, by virtue of the modern perfection in technical details of examination and the marked improvement in instrumental construction. It must be remembered, however, that regardless of the type of instrument employed and the dexterity of the operator, only a small portion of the urethral mucosa can be exposed to visual inspection at one time, and reconstruction of a correct pathological picture from exposures which are necessarily fragmentary is sometimes exceedingly difficult.

To the experienced urologist the technique of both anterior and posterior urethroscopy is comparatively simple. Where there exists no pathologic narrowing of the canal from any cause, such as inflammatory exudative stenosis, the presence of polypoid excrescences, etc., the tube selected should be the largest which can be inserted through the meatus externus without causing pain; if the meatal opening is abnormally small, meatotomy should be performed before attempting introduction of the endoscope. The degree of dilatition which may sometimes be secured by careful introduction of the urethroscope in urethræ which seem abnormally small is surprising. If there be any considerable degree of pathological stenosis, preliminary dilatation may be necessary before the endoscope can be successfully introduced. If the urethra appears unduly sensitive local anesthesia is advisable. No instrument should be inserted which has not been carefully sterilized and properly lubricated.

After cleansing the glans penis and meatus externus with a mild antiseptic solution, with the patient in the lithotomy posture, the urethroscope is gently and very gradually introduced, thus avoiding all possibility of inflicting injury upon the delicate urethral mucosa. Where insertion seems impossible because of the limited caliber of the urethra or the presence of stenosis, the canal must be gradually dilated until it will admit at least a No. 24 F. sound. After the urethroscope has entered the canal, the obturator is removed, the lamp being then adjusted and inserted, and the operator is ready to begin his inspection as the instrument is slowly withdrawn. If the patient appears nervous and excited, and especially if the urethra is sensitive to instrumentation, it is advisable to secure anesthesia by the introduction of a two per cent. solution of novocaine. While cocaine is often used for this purpose, its application may be attended by danger because of the pronounced absorbing power of the urethral mucosa. Fatal absorption has occurred from the intraurethral application of strong solutions of cocaine.

As the urethroscope is gradually withdrawn the operator visually inspects the entire length of the urethral lumen. "Urethroscopy is a diagnostic means of especial value in chronic inflammatory states of the posterior urethra, pre-eminently so in glandular inflammatory involvement of the latter." (Aronstam.) Where there is any considerable secretion, the field under inspection must be frequently cleansed with pledgets of cotton upon a suitable instrument introduced through the endoscopic tube. Even the slight hemorrhage which may occur from contact of the instrument with existing urethral erosions may seriously obscure the area being scrutinized, and care must always be exercised in keeping the field properly cleansed.

An adequate appreciation of the pathological urethra is impossible without a thorough understanding of its normal appearance. In color the normal urethral mucosa varies from yellowish-white (or pale-pink) near the meatus to deep red in the posterior portion. The luster is practically uniform throughout; the upper surface is moist and glossy, being smooth in that portion near the glans. The deeper walls, however, are in contact with each other in the form of longitudinal folds; when the endoscope is introdhced these folds present a radiate appearance, the radiations varying somewhat according to the size of the tube, i. e., the smaller the instrument the greater the number of plications, and vice versa. Striations varying in color from pink to deep red (from the mucosal blood vessels) may be noted in the spaces between the folds; these striations are not really "straight lines," as they may be obliterated by contact with the tube; pressure exerted in different directions may also produce areas of anemia and hyperemia; in some instances the mucosal capillary network may be plainly noted. The crypts of Morgagni may be observed upon the upper wall as longitudinal depressions without evidence of secretion, the orifices being usually invisible in the normal urethra; the openings of the ducts of Cowper's glands are sometimes visible in the lower wall of the bulbous portion; the colliculus seminalis is always in evidence.

The endoscopic picture is funnel-shaped, the base is formed by the edge of the tube, the apex being further inward, the sides consisting of the urethral walls. The extent of the area under visual inspection depends largely upon the size of the instrument in comparison to the urethral caliber. It must not be forgotten that the meatus represents the narrowest portion, and that the lumen may elsewhere vary considerably in diameter; thus the appearance of the mucosa may differ at various points. The bulbous section is described as "a folded floor and a horizontally tense roof;" the pendulus portion shows the most accurately developed funnel-like configuration of the mucosa, and five to six concentric folds may be noted disappearing distally into the so-called closed "central figure;" the surface of the glandular portion is more even and the folds less marked, the center being always slightly patulous.

The importance of endoscopy in pathology implicating eithe the anterior or posterior urethra cannot be overestimated. In the acute stages of inflammatory disease, however, urethral instrumentation is distinctly contra-indicated and much harm might result from its employment. It is in certain types of chronic urethral lesions that the urethroscope probably has its greatest field of usefulness, (a) to insure diagnostic accuracy, (b) to permit application of appropriate therapeutic agents and surgical procedures, (c) to determine the progress or retrogression of the lesion as a result of treatment, and (d) to ascertain whether or not a permanent cure has been effected. The views of other observers to the contrary notwithstanding, experience suggests that belief in the existence of so-called symptomless urethral lesions must represent either a fanciful delusion or be a confession of diagnostic and pathologic ignorance upon the part of the clinician. In justice to those who hold different opinions, however, it is but fair to add that the symptoms resulting from chronic urethral pathology are oftentimes referred to other anatomical situations. No doubt in many cases so-called chronic sexual neurasthenia, obscure neuroses, etc., owe their origin to urethral infiltrations, contractions, erosions or other lesions representing logical sequelæ of chronic inflammation, or from other pathology within the anterior or posterior urethra including the colliculus, the prostate and the urethro-vesical orifice. Numerous instances might be cited where stenosis so moderate in degree as to be unsuspected by the patient was responsible for the production and maintenance of the varied symptom-complex of neurasthenia with total sexual impotence extending over considerable periods of time, in which adequate treatment caused prompt subsidence of the neurotic manifestations with return of former virility. In such cases the urethroscope is not only an especially valuable diagnostic measure, but its importance in preventing further sequelæ by permitting recognition and removal of the existing pathology has hitherto been insufficiently emphasized.

Owing to the intimate connection of the posterior urethra (particularly the prostate and colliculus) with the sympathetic nervous system, and through the latter with certain important organs of the body, the symptom-complex of posterior urethral pathology is exceedingly variable. In many instances the pain attending posterior urethral lesions is referred to the legs, to the back, etc.; and oftentimes the sufferer is erroneously treated for rectal or allied pathology, with constant aggravation rather than improvement in existing symptoms. Much emphasis has recently been placed upon the relationship between posterior urethritis (particularly prostatitis and coliculitis) and sexual neurasthenia, which in many instances has disappeared by suitable applications through the endoscope to the colliculus (Englander).

To describe the minutiæ in connection with the various types of urethral pathology clearly recognizable by visual inspection through the modern endoscope would unduly prolong this paper, therefore, only brief outlines can be considered. The endoscopic picture of the pathological urethra furnishes a decided contrast to the normal. The mucosa may be anemic, hyperemic, inflamed, thickened, edematous and infiltrated; granular circumscribed areas of black or dark-red are sometimes noted; as a result of infiltration and development of scar tissue the epithelium is destroyed and the normal luster of the mucosa disappears; thickening prevents the formation of normal appearing folds, and the striations are less marked or entirely lacking; when Littre's glands (normally invisible) participate in the pathology they appear as small, rounded depressions, with slit-like openings and everted dark-red edges; in the rare affection known as psoriasis mucosæ (Kollman-Oberlander) the urethral surface is covered with lusterless, flat, whitened, firmly adherent patches.

The various types of urethra erosions, ulcerations, tumor formations, diverticula, polypi, cysts, etc.; are easily recognized and differentiated by visual inspection through the modern endoscope, as is also the so-called "stricture of large caliber" (Otis) to which attention has already been called. It must not be forgotten that urethral instrumentation is contra-indicated in the presence of acute inflammatory lesions also in tuberculosis and carcinomatous ulcerations, although in the latter endoscopy may be sometimes permissible as an aid to accuracy in diagnosis.

REFERENCES

Aronstam: UROLOGIC AND CUTANEOUS REVIEW, September, 1915.

Casper: "Genito-Urinary Diseases."

Englander: UROLOGIC AND CUTANEOUS REVIEW March, 1914.

Gordon: AMERICAN JOURNAL OF UROLOGY, September, 1915.

Guiteras: "Urology," 1912, Vol. I.

Oberlander: AMERICAN JOURNAL OF UROLOGY, January, 1913.

Ross: UROLOGIC AND CUTANEOUS REVIEW March, 1914.

Rusby: Ref. Handbook of the Med. Sci., Vol. II.

White-Martin: "Genito-Urinary and Venereal Diseases."

Wossidlo: ZEIT F. UROL., 1914; AMN. J. OF UROLOGY August, 1914.

Wossidlo: SUPPLEMENT TO U. AND C. REVIEW, January, 1913.

---

### THE DOCTOR'S HEAVEN.

I dreamed that I was talking,
With a doctor, old and gray.
Who told me of a dream he had,
I think 'twas New Year's Day.
While snoozing in his office,
The vision came to view,
For he saw an angel enter,
Dressed in garments white and new.
Said the angel, "I'm from heaven,
Peter sent me away down
To bring you up to glory,
And put on you a golden crown.
You've been a friend to every one,

And worked both night and day,

You've doctored many thousands,

And from few received your pay,

So we want you up in glory,

For you have labored hard,

And the good Lord is preparing,

Your eternal just reward."

Then the angel and the doctor,

Started up to glory's gate,

But when passing close to Hades,

The angel whispered "Wait."

I've a place I want to show you,

It's the hottest in all hell,

Where the ones who never paid you,

In torment must always dwell."

And behold the doctor saw there,

His old patients by the score:

Then grabbing up a chair and fan,

He wished for nothing more.

Just content to sit and watch them,

As they sizzle, singe and burn,

And his eyes would rest on others,

Whichever way they'd turn.

Said the angle, "Come on, doctor,

There the pearly gates I see,"

But the doctor only murmured,

"This is heaven enough for me."—Anonymous.

—Sandersville, Ga., "Progress."

### THE NEW INDEPENDENCE; MADE IN AMERICA; PATRIOTISM IN THE MEDICAL PROFESSION.

DR. G. HENRI BOGART, PARIS, ILLINOIS.

(TEXAS MEDICAL JOURNAL.)

When the great war first commenced the medical man wondered where he was to obtain the customary drugs; now the restrictions of commerce has put the hobble on the other foot and the hemmed-in belligerants—and lots of other folks—are wondering about the food supply.

The whole world has been crying for the moon, has been turning to the absent distance for the glamor, passing up the "something just as good" or better at home.

The Martians may be supposed to gaze down at their evening star, our earth, in its wondrous purity of white beauty, unthinking of the bottomless, spring-thawed roads of Illinois, or the discoveries of the vice commissions; botches on its silvery sheen, for that star is our earth, and other drawbacks.

I have ridden through the woodlands of Indiana, when the hillsides glowed with acres of the purple splendor of the digitalis, while the farmer cursed it, the stagger weed that poisoned his cattle, or compelled him to forego the use of the lush pasturage, and then stopped at the drug store to purchase digitalis "made in Germany." I have seen the village physician paying a laborer to grub the "Wandering Milkweed" from his pasture lot, and at the same time prescribing the alkaloid from those same laboratories across the sea, the while his father had learned the virtues of pleurisy root from the older botanic school.

No, I do not mean that we shall go back, and with shears and shovel gather our sources for infusion and decoction.

America has splendid laboratories with well won reputation for alkaloids, specific tinctures and extracts second to none; we have unlimited fields of crude material, the plants and minerals in abundance, either neglected or harvested for shipment to the laboratories across the ocean, on whom we have come to depend.

We prate about our independence, and then depend, slavishly, upon those others, while neglecting our own manufacturers. We cannot expect our own chemists to flourish unless we support them, for we read of that which grows by that it doth feed them.

I have trellises of roses about my home, vines that yield a wealth of perfumed beauty, that would not be, did I not furnish the support and loving care for their growing.

As a duty, as pure patriotism, we should declare a new era of independence and we need not wait until the Fourth of July, either, rather

"Act, act in the living present."

We need no Congress, no Liberty Hall; we need just you, and you, and you, all pulling together for the sustenance of home remedies.

I have been amused and disgusted at the prominence given the "Twilight Sleep," with all the twaddle and rigmarole of the lay press, exploiting that something new that drew the Greeks about St. Paul, on Mars Hill, when right here in America we have had for years, a more dependable preparation, in tablet form on the market, permanently dispensed, and with an abundant free literature on the subject, and nothing has been said about it, only in a business way to the profession; you could not have driven a newspaper or a magazine reporter to have peeped through the portals of the possibilities of this "Made in America" release t he pangs and terrors of childbirth, for the story would have been commercial, would have lacked the twang and novelty of importation, and would have violated the ethics of publication, by advertising something as a news feature. This publication, with gratis literature, has been before the American public, but met silence, while its more clumsy foreign substitute was boomed as a sensation, a scoop.

There is the axiom of supply and demand that answers the question as to the ability of our chemists and pharmacists to supply all the skill and products we need.

We spend many millions, annually, on scientific education, and then calmly close our eyes and leave the men and women whom we have trained, to potter and piddle, for want of a demand for their services, that shall sharpent their faculties and reward their efforts; throttling their ambitions instead of spurring them on.

Here is one example of what incentive will do.

Edison was using tons of carbolic acid each week, when the war stopped his supply. He had been told that our coals would not furnish it. He did not shut down his works; he investigated making of a synthetic product, got busy, and within a month was making a superior product, cheaper than the foreign chemist had produced it.

America can furnish as good and better chemicals than can the foreigner if the rank and file support the demand. Our chemists and pharmacists are able, willing and anxious to furnish for our utmost need if we give the opportunity. If you are not satisfied with the reactions of a remedy; if you need something that it lacks, tell your manufacturer, write him candidly, take him into your confidence; he is more than willing to co-operate.

If you know what you want, he will be more than willing to meet you more than half way, to know and to solve your difficulties.

Be patriotic in the highest sense, that of the upbuilding of your own country, which is better than hurting and destroying your neighbor nation.

Make yourself one of the signers of the new Declaration of Independent and do it now; having done it, keep on doing it.

---

### AMERICAN PROCTOLOGIC SOCIETY, DETROIT, MICH. PROGRAM, COMMENCING JUNE 12, 1916.

Executive Council meets at 11 A. M.

First Regular Session at 2 P. M.

Annual Address of the President—Subject: Why Proctology has been made a Specialty. T. Chittenden Hill, Boston, Mass.

PAPERS.

1—A Review of Proctologic Literature for 1915. Samuel T. Earle, Baltimore, Md.

2—Post-Operative Treatment in Rectal Surgery. Wm. H. Stauffer, St. Louis, Mo.

3—Ano-rectal Injuries. Samuel G. Gant, New York City, N. Y.

4—Some Observations on Hernia in Relation to Intestinal Stasis. Wm. M. Beach, Pittsburg, Pa.

5—Intestinal Symptoms due to Achylia Gastrica. Alois B. Graham, Indianapolis, Ind.

6—Non-Specific Ulceration of the Rectum and Anus, with Report of a Case of Anal Herpes Zoster. Lewis H. Adler, Jr., Philadelphia, Pa.

7—Malignant Transfomation of Benign Growths. Frank C. Yeomans, New York City, N. Y.

8—Acute Angulation and Flexure of the Sigmoid as a Causative Factor in Epilepsy; Report of nine new Cases with four Recoveries. Wm. H. Axtell, Bellingham, Wash.

9—The Vaccine Treatment of Pruritus Ani. W. H. Kiger, Los Angeles, Cal.

10—Report of Experience with the Vaccine Treatment of Pruritus Ani. Louis J. Hirschman, Detroit, Mich.

11—Posture as an Etiologic Factor in Splanchnoptosis. Rolla Camden, Parkersburg, W. Va.

12—Photography for Record and Teaching; Lantern Slide Demonstrations. Collier F. Martin, Philadelphia, Pa.

13—The Present Status of Operations for Carcinoma of the Rectum and Lower Third of the Sigmoid. Samuel T. Earle, Baltimore, Md.

14—Observations on Fissure of the Anus. Rollin H. Barnes, St. Louis, Mo.

15—The Treatment of Hemorrhoids by a New Method. E. H. Terrell, Richmond, Va.

16—The Relation of Colonic Disease to the Kinetic System. James A. MacMillan, Detroit, Mich.

17—The Consideration of Rectal and Colonic Disease in Life Insurance Examinations. Alfred J. Zobel, San Francisco, Cal.

18—Spasmodic Stricture of the Rectum. Louis J. Krouse, Cincinnati, Ohio.

19—Some Important Pathological Conditions found About the Rectal Outlet. Lantern Slide Demonstration. Granville S. Hanes, Louisville, Ky.

20—The Relation of the Roentgenologist to the Proctologist. Walter I. Le Fevre, Cleveland, Ohio.

21—Syphilis of the Rectum. G. Milton Linthicum, Baltimore, Md.

22—Position for Sigmoidoscopic Work. Donly C. Hawley, Burlington, Vt.

23—Sixth Report on the Treatment of Pruritus Ani by Autogenous Vaccines. Dwight H. Murray, Syracuse, N. Y.

24—Gangrenous Hemorrhoids; Reports of Cases. John L. Jelks, Memphis, Tenn.

